# Piezoelectric Property of Electrospun PVDF Nanofibers as Linking Tips of Artificial-Hair-Cell Structures in Cochlea

**DOI:** 10.3390/nano12091466

**Published:** 2022-04-26

**Authors:** Rana Sabouni Tabari, Yu Chen, Kunyapat Thummavichai, Yan Zhang, Zakaria Saadi, Ana I. S. Neves, Yongde Xia, Yanqiu Zhu

**Affiliations:** 1 Department of Engineering, University of Exeter, Exeter EX4 4SB, UK; rs683@exeter.ac.uk (R.S.T.); kt302@exeter.ac.uk (K.T.); z.saadi@exeter.ac.uk (Z.S.); a.neves@exeter.ac.uk (A.I.S.N.); y.xia@exeter.ac.uk (Y.X.); 2 State Key Laboratory of Powder Metallurgy, Central South University, Changsha 410083, China; 219120@csu.edu.cn

**Keywords:** electrospun PVDF fiber, tip links in cochlea, piezoelectric properties, hearing loss

## Abstract

The death of hair cells and damage of natural tip links is one of the main causes of hearing-loss disability, and the development of an advanced artificial hearing aid holds the key to assisting those suffering from hearing loss. This study demonstrates the potential of using electrospun polyvinylidene fluoride (PVDF) fibers to serve as the artificial tip links, for long-term hearing-aid-device development based on their piezoelectric properties. We have shown that the electrospun PVDF-fiber web, consisting of fibers ranging from 30–220 nm in diameter with high β-phase content, possesses the high piezoresponse of 170 mV. Analyses based on combined characterization methods including SEM, TEM, XRD, FTIR, Raman, DSC, XPS, PFM and piezoelectricity have confirmed that an optimized value of 15 wt.% PVDF could act as an effective candidate for a tip-link connector in a vibration-frequency prototype. Based on this easily reproducible electrospinning technique and the multifunctionalities of the resulting PVDF fibers, this fundamental study may shed light on the bio-inspired design of artificial, self-powered, high performance, hair-cell-like sensors in cochlea to tackle the hearing loss issue.

## 1. Introduction

Hearing loss, as one of the most common disabilities, will directly affect 2.5 billion people in the world, which accounts for more than 6.1% of the global population by 2050 [1]. Modern hearing-aid techniques have been rapidly developed, but the requirement for permanent use of these devices makes the users uncomfortable. Furthermore, external batteries are required to power the device for everyday use, which is inconvenient. The restoration of hearing loss involves the re-connection of hair cells via tip links, which act as the mechanoelectrical transducer (MET) to convert the mechanical motion of the hair cells caused by sound (acoustic vibration) into electric signals [2].

So far, artificial-hair-cell sensors offer new directions to restore hearing by creating artificial hair cells using Si-based micro and nanofabrication technologies, and current artificial tip links are made of hybrid semiconductors, metals, oxides and polymers, based on different principles such as electrostatic, piezoresistive or thermal sensing [3,4,5]. The tip-link connectors serve as transducers to measure the mechanical displacements. By applying a mechanical pressure, the tip links become deformed and measurable electrical potentials are generated. A further advanced concept is the self-powered, implantable artificial-hair-cell sensor, which is based on the piezoelectric properties of a material, and converts the applied stress caused by sound energy into electricity to power the device [6]. Recent piezoelectric hair-cell developments include lead zirconate titanate (PZT) thin film, Pyrex glass wafer, Ti/Pt electrode, polyvinylidene fluoride-trifluoroethylene (PVDF–TrFE) piezoelectric films, which have been fabricated for the air-fluid type with lower voltage [7,8,9].

As an attractive piezoelectric material, polyvinylidene fluoride (PVDF) exhibits a high piezoelectric coefficient, good flexibility, toughness, and excellent biocompatibility; it has shown advantages in responding to a great range of motion and high acoustic impedance, and it is easy to process. Recent work has shown that an artificial PVDF–TrFE membrane cochlear epithelium sensor in a Guinea pig cochlea is capable of generating an electrical output from 0.14 to 5.88 mV, which is a range that has the potential for omitting the use of an external power amplifier and/or battery [10]. It is therefore feasible to develop the next generation of self-powered intelligent hearing aids by using PVDF without the need for any external power sources, leading to the omission of battery usage, which is essential to the patient in some cases [11,12,13,14,15,16,17]. Early attempts at creating self-powered sensors to detect mechanical vibrations have been documented [18,19,20]. However, the functionalities of PVDF depend on various factors such as fabrication, processing, crystallinity, geometry, etc., which need to be thoroughly understood prior to attempting to incorporate them as tip links in a hearing sensor. Therefore, to fabricate PVDF tip links with an optimal structural, the phase type and functional features are critical factors in the construction of an artificial hearing-aid device. Recent advances in PVDF materials have proven that a PVDF-based implantable cochlear implant (CI) was feasible for compressive pressure measurement; however, the amplification capacity needs to be improved.

Aiming to develop a self-powered intelligent hearing-aid device using artificial hair cells, in this paper, we demonstrate the full potentials of PVDF as the tip-link connector by using the electrospinning technique to generate β-phase-dominated continuous nanofibers that are piezoeffective [21,22]. As a result, the interesting piezoelectric PVDF fibers could be utilized to deliver an electrical signal to an artificial cochlear implant. The morphology, phase structures, and piezoelectric functionality of the PVDF fibers produced by electrospinning are thoroughly evaluated, and a prototype of the device using electrospun PVDF nanofibers as the tip-link connectors for an intelligent hearing-aid device is demonstrated. The piezoelectric property of the electrospun PVDF fibers at the nanoscale is effectively characterized by piezoresponse force microscopy (PFM), which is a non-invasive method for determining the piezoelectric response of ferroelectric samples [18,19].

**Figure 1:**
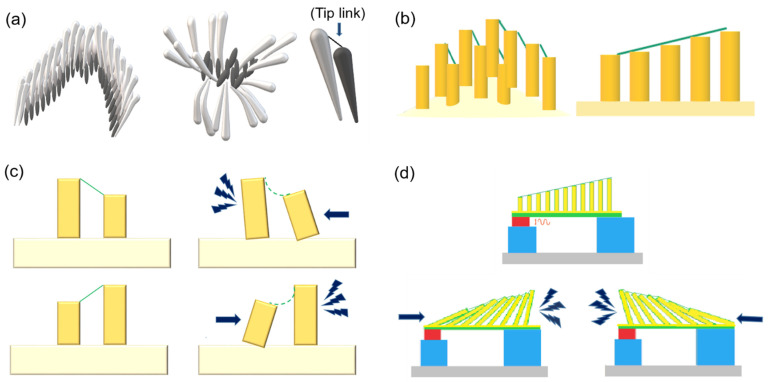
(**a**) Natural hair cells in the inner ear and broken hair cells; (**b**) a 3D model of artificial hair cells including polydimethylsiloxane (PDMS) pillars and electrospun PVDF tip link; (**c**) a cross-section model of neighborhood elastic pillars connected with the electrospun PVDF nanofibers mimicking the tip links in the cochlea and (**d**) a 3D schematic of hair-cell-like sensor device.

Figure 1a illustrates the natural hair cells in cochlea, and Figure 1b,c are schematics of the PVDF artificial tip links highlighted in this study, which link the artificial hair cells in the prototype. An ideal tip link should be made with an extremely sensitive transduction mechanism to couple mechanical input to electrical output. Figure 1d is the proposed artificial-hair-cell-like sensor including tip links as connectors of artificial pillars.

## 2. Materials and Methods

### 2.1. Materials

Poly(vinylidene fluoride) (PVDF) powder with a mean particle size of 20 µm and an average molecular weight of 524,000 g mol^−1^, N,N-dimethylformamide (DMF) anhydrous, 99.8%, and acetone, polyethylene terephthalate (PET) sheet, were purchased from Goodfellow (Huntingdon, UK). The 3M 1181 conductive copper tape of 50 mm width used for the triboelectricity was bought from RS (Plymouth, UK).

### 2.2. Solution Preparation

Different concentrations of PVDF polymeric solutions of 15, 16.5, and 18 wt% were prepared by adding PVDF powder measured with the aid of ALJ 160-4AM scales (accuracy category (I): e > 0.001 g) into 10 mL DMF/acetone (3/7) volume ratio of solution, with the assistance of a magnetic stirring hot plate (Yizerel brand with the error of temperature measurement: <0.5%) at 600 rpm at 70 °C for overnight. The homogenous and viscous solutions were the stock solutions for all experiments.

### 2.3. Electrospinning Methodology Application

**Figure 2:**
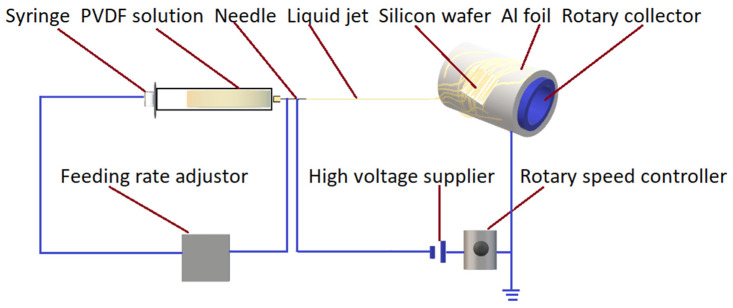
Schematic of the electrospinning set-up.

The electrospinning experimental set up is illustrated in Figure 2, which consisted of a feeding pump, high-voltage power supply, needle, collector, and syringe with a PVDF fluid. Herein, the samples were collected in ambient temperature, the outer diameter of the syringe containing solutions was 13.7 mm and the needle dimension attached to the syringe was size 18 G with a dead volume of 25.4 mm. The speed of the collector was 1200 rpm. The rotary-drum collector (100 mm in diameter) was covered by an Al foil for the easy collection of nanofibers.

Prior to spinning, each stock polymer solution was fed into the needle. Different parameter sets, including the differences in the concentrations (sample A: 15 wt%, sample B: 16.5 wt% and sample C: 18 wt%), Figure S1, Table S1,the applying voltages (sample D: 10 kV, sample E: 15 kV and sample F: 20 kV), Figure S2, Table S2, the distance between the needle and the collector (sample G: 10 cm, sample H: 15 cm and sample I: 20 cm), Figure S3, Table S3, feeding rates (sample J: 1 mL, sample K: 3 mL and sample L: 5 mL), Figure S4, Table S4 and rotary speeds of the collector (sample M: 400 rpm, sample N: 800 rpm and sample O: 1200 rpm), Figure S5, Table S5 were tested (Supplementary Materials), and only the optimized results are reported in this context. The rotary-drum collector can help to create aligned fibers in large areas. For characterization purposes, some fibers were collected on a stationary Si wafer or Al foil fixed on the collector. The effectiveness of different parameters on the fabrication of the thinnest electrospun fibers in terms of reaching the highest β phase formation was investigated by a sequence of experiments in this study; however, a multivariate design to optimize the β-phase formation by introducing a chemometric approach would be more appropriate towards this goal.

### 2.4. Characterization

The structure and morphology of all samples were examined using a scanning electron microscope (SEM, TESCAN VEGA3 model, Brno-Kohoutovice, Czech Republic), operated at 10 kV and a transmission electron microscope (TEM, JEOL 2100, Tokyo, Japan) operated at 200 kV voltage. The SEM samples were preliminarily coated with a thin chromium layer (about 5 nm) by using the sputtering technique for high-resolution imaging. Moreover, XRD measurements were recorded using a (Bruker D8, Oxford, UK) Advanced diffractometer using a Cu radiation source, operated at 40 kV and 40 mA, scanned within the 2θ range from 10 to 70°.

FTIR spectroscopic analysis was conducted using a (Bruker Optics Tensor-27 instrument, Bruker cooperation, Coventry, UK) and infrared (IR) absorbance spectra were obtained between 700 and 1400 cm^−1^ at a resolution of 4 cm^−1^ using 20 co-added scans. A total of 15 mg of each sample was mixed with 150 mg spectroscopic grade KBr in an agate mortar and compressed into small tablets. Tablets were formed by pressing the resulting mixtures under 5 tons for 2 min and heated at 60 °C for 2 h. Each tablet was placed into an attachment for the FTIR analysis.

For the DSC evaluation, a (Mettler Toledo, DSC 1 STARe system, Leicester, UK) with intracooler, DSC823e was applied. All of the electrospun nanofibers were heated at a rate of 15 °C min^−1^ from 20 to 200 °C, under a N_2_ flow of 100 mL min^−1^.

XPS measurements were carried out on a (Kratos AXIS Ultra HAS spectrometer, Manchester, UK) with a monochromatized Al–Kr X-ray source (10 mA emission current and 15 kV anode potential operation). The spun fiber membranes were mounted on the standard sample studs by means of double-sided adhesive tapes. Raman spectroscopy was conducted using a Renishaw benchtop Raman system, with 532 nm excitation wavelength and 2400 L mm^−1^ grating, to acquire the Raman spectra of all samples.

Piezoresponse force microscopy (PFM) was conducted on an atomic-force microscope (NanoManTM VS, Veeco company, New York, NY, USA) in contact mode using a conductive Pt/Ir-coated Si cantilever (SCM-PIT) for the piezoelectric-property characterization. The electrospun PVDF samples were thermally etched and shrunk to 0.2 mm in length before the measurement. A voltage from −12.5 to 12.5 V was applied while superimposed on an AC modulation voltage during polarization switching. This assessment is needed for its electro-based transduction mechanism known as the piezoelectric effect for the fabricated fibrous mat of the electrospun PVDF fibers.

For the piezoelectric nanogenerator (PENG) characterization of the fabricated device, the vertical contact-separation mode of a linear voice coil actuator was used, measured at room temperatures varying between 21.7–24 °C and a humidity between 38–45% for 2000 cycles at a frequency of 1 Hz and a consistent value of force applied, equal to 40 N. This can be helpful for movement-simulation purposes of electrospun nanofibers replicating tip links of hair cells. Additionally, such force allows maximum surface contact area to maximize the potential difference and the current. The order of the materials and their positions was a copper tape of 30 × 30 mm^2^ electrospun with PVDF nanofibers moving toward the other surface of a PET sheet attached to a copper-sheet surface with similar dimensions as illustrated in Figure S6. The mean stable outputs for 5 peaks were measured as the voltage and current outputs.

## 3. Results and Discussion

### 3.1. Optimal Parameters for Electrospun PVDF Fibers

**Figure 3:**
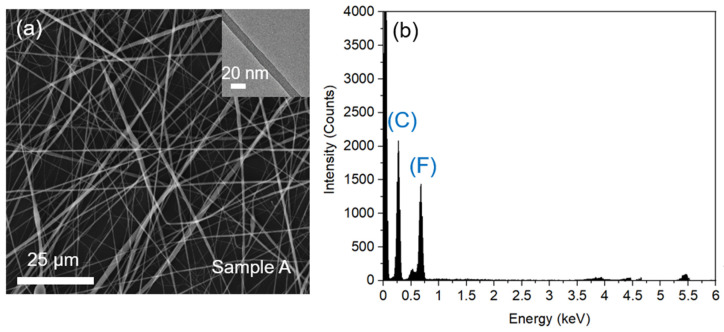
(**a**) SEM and TEM image of 15 wt.% electrospun PVDF fibers with the average size of 20–220 nm (standard deviation: 51.6 nm) and (**b**) EDS spectroscopy of sample A.

In order to achieve PVDF nanofibers with a controllable morphology and uniform diameter, the precursor concentrations, feeding rate, voltage value, distance between the needle and the drum collector, and the rotary speed of the drum collector were tested and optimized. The results are presented in the Supporting Materials. Under a set of ideal parameters of 15 wt% precursor concentration, 20 kV operating voltage, 15 cm distance, 3 mL h^−1^ feeding rate at 1200 rpm collector speed, nanofibers with diameters ranging from 20–220 nm can be controllably created, as shown in Figure 3a. The structures and properties of the generated PVDF fibers were fully analyzed via different techniques and their application as artificial tip links was tested.

### 3.2. Structural and Phase Features of the PVDF Nanofibers

**Figure 4:**
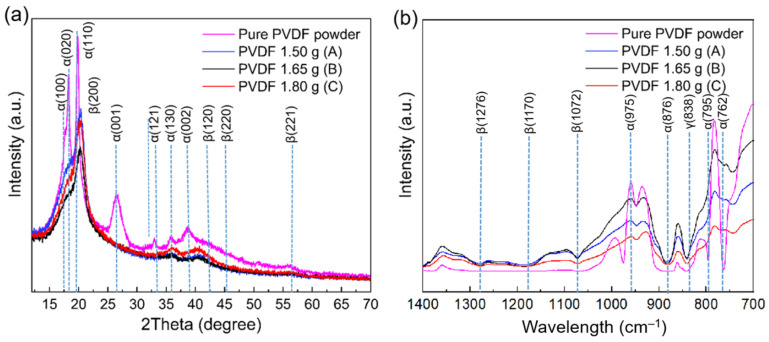
(**a**) XRD profiles of pure PVDF powder and electrospun fibers of different concentrations of PVDF and (**b**) FTIR spectrograph for PVDF powder including α- and γ-phases, and three different concentrations of electrospun PVDF nanofibers including β-phase.

The X-ray-diffraction (XRD) profiles shown in Figure 4a present a comparison between the pure semi-crystalline PVDF powder and the electrospun nanofibers that were spun from three different PVDF concentrations in the same 10 mL solution, named as samples of A (15 wt.%), B (16.5 wt.%) and C (18 wt.%). The spun fibers exhibited one intense peak at 20.8° (200), two weak shoulder peaks at 36.6° (120) and 39° (220), and a peak at 56.9° (221), which correspond to the β-phase of the electrospun PVDF fibers [22]. The pure PVDF powder exhibited peaks at 18.4° (020), 19.8° (110), 26.4° (001), 33.2° (121), 35.9° (130) and 38.8° (002), which are indexed to the α- and γ-phases, but not the β-phase [23,24,25,26,27]. A further comparison of the relative intensity ratios of diffraction peaks at 2θ of 20.8° and 38.8° as indicators for the β-phase content yielded values of 4.73, 4.30 and 3.87 for sample A, B and C, respectively. Interestingly, the β-phase relative intensity at these 2-thetas for sample B was higher than sample C but lower than that of sample A. The high level of β-phase for sample A can be attributed to the better interactions between PVDF and the solvent at this concentration. As the solvent for this study was DMF, the dipolar interfaces between C=O and CH2-CF2 in the solvent and PVDF in addition to the other weak hydrogen bonding such as C=O…..H-C will make the β-phase appearance possible [28,29]. For sample A, the sharper β-phase peak is a consequence of the electrospinning that had the highest electric-field burst of speed in the stretching of the polymer jet. Therefore, the most suitable concentration for achieving the highest β-phase for optimal piezoelectricity properties is 15 wt.%.

The bonding information of the electrospun PVDF fibers and pure PVDF powder were measured by FTIR and the results are shown in Figure 4b. In the fiber samples, transmittance bands at 840, 1,072, 1,170 and 1,276 cm^−1^ correspond to the β-phase, while the other bands at 762, 795, 876, and 975 cm^−1^ are due to the α-phase in the pure PVDF powder. The powder sample displayed a band at 838 cm^−1^ for the γ-phase, which is close to the value of the β-phase band in the Fourier-transform infrared (FTIR) results. The relative intensity ratio of fixed wavelengths between 840 cm^−1^ and 1,170 cm^−1^ for β-phase was equal to 0.95 for sample A, 0.91 for sample B, and 0.90 for sample C, which provides further evidence that sample A contains the highest β-phase content of 89%, as shown in Figure S7 and Table S6. The quantification of the β-phase by the FTIR results is derived from the Lambert–Beer law, and the most promising candidate for achieving the highest piezoelectric properties is sample A with the maximum value of F(β) = 0.89.

Among all the PVDF phases (α, β, γ, δ and ε), the piezo-, ferro- and pyroelectric effects of the β-phase are stronger than those of the other phases [30,31,32,33,34,35]. Furthermore, the β-phase possesses a relatively high dipole moment due to the polymer chain arrangement in a single line of hydrogen and fluorine, which endows it with high piezoelectric coefficient for energy-storage devices [36]. For the β-phase, due to the trans-planar zigzag (TTTT) structure in the main chain, there is a dipole moment per unit cell which is the highest, leading to the highest piezoelectric properties. These attractive properties of β-phase PVDF polymers make them promising for energy-conversion applications, such as electromechanical actuators, micro-electromechanical devices, and energy harvesters [37].

**Figure 5:**
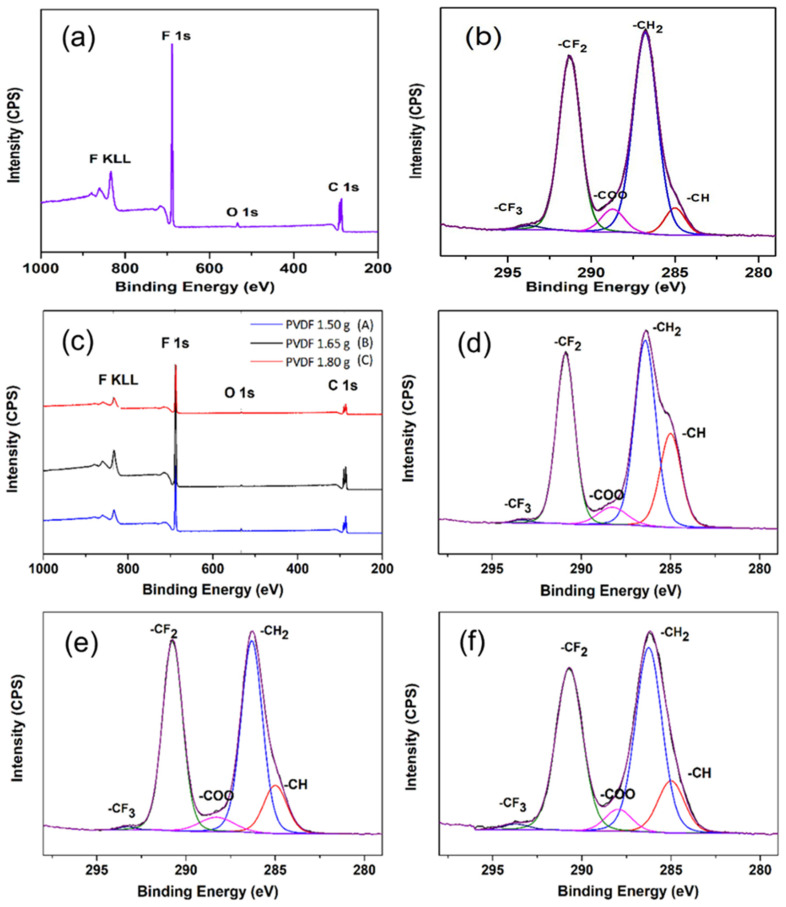
(**a**,**b**) XPS spectra of pure PVDF powder and (**c**–**f**) XPS spectra of electrospun PVDF nanofibers for samples A, B and C.

X-ray photoelectron spectroscopy (XPS) was used to analyze the elemental composition and chemical state of the raw PVDF powder and different fiber samples (Figure 5). The peaks of F 1s (~688 eV) and C 1s (~286 eV) regions, as well as F KLL Auger transition were clearly observed in the XPS survey spectrum of all PVDF samples (Figure 5a,c) [38]. The O 1s (~532 eV) of all samples was also observed in low intensity in the survey spectrum. The peak of C 1s of all samples were resolved by the curve fitting of the five peaks that corresponded to the elements and groups directly boned to carbon atoms (Figure 5b,d–f).

Two main peaks at about 286 and 290 eV are likely to have originated from the –CH_2_– and –CF_2_– components in PVDF, respectively [39]. The peaks at about 285 and 288 eV correspond to –CH– and the carbon atom in a strong electron-withdrawing environment (for example, in the ester group, amide group, or bonded to a withdrawing atom such as N or O, assigned to –COO), respectively [40]. A small peak at the highest binding energy of about 293 eV corresponds to –CF_3_– groups, as seen in perfluorinated ethers [41]. Based on the list of the element compositions of both pristine PVDF and PVDF fibers (Table 1), the O 1s/C 1s and F 1s/C 1s ratios of all samples were found to be at the range of 0.016–0.031 and 0.65–0.8, respectively, which fall within the range of PVDF from the literature [42]. The variation of each element might be subject to contamination during the preparation process or/and dehydrohalogenation of PVDF [38,39,40,41]. The percentage of each component of the C 1s spectrum is also summarized in Table 2. The ratios of –CH_2_– and –CF_2_– components are 1.3, 1.24, 1.1 and 1.14 for the pristine PVDF and samples A, B and C, respectively. The increasing percentage of CH from the fiber samples could be affected by the dimethylformamide (DMF) that was used as the solvent during the fiber preparation. The XPS results confirm that the PVDF was successfully prepared in the fiber form, and the fiber samples remained similar to the pristine PVDF in terms of their elemental composition and chemical states.

The C:H:F ratio obtained from the spectrum after curve fitting is 1:1:0.8, respectively, which is in good agreement with the ratio obtained from the EDS result shown in Figure 3b. Additionally, this result further agrees with the EDS spectroscopy result of the electrospun fibers, as shown in Figure 3b.

**Figure 6:**
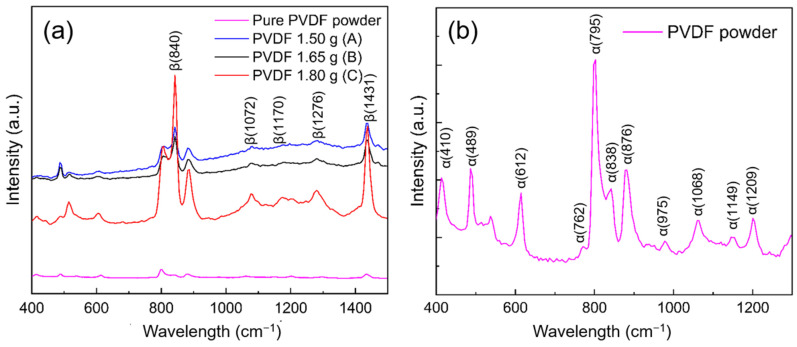
(**a**) Raman spectra of electrospun PVDF nanofibers including samples A, B and C and (**b**) pure PVDF powder.

According to the Raman spectroscopy analysis (Figure 6), all phases of fabricated electrospun fibers as well as the powder sample could be distinguished. In the powder sample, both α- and γ-phases existed, whilst in the electrospun fibers, the major peaks were assigned to the β-phase. The α-phase in the powder sample exhibited shifts at 410, 489, 612, 762, 876, 975, 1068, 1149 and 1209 cm^−1^ and the shift of the γ phase appeared at 838 cm^−1^. The intensities of the β-phase mode in all samples were relatively higher compared with those of the α-phase for the commercial PVDF powder sample. For the electrospun fibers, the Raman shifts of the β-phase were situated at 795, 840, 1072, 1276 and 1431 cm^−1^, which are in accordance with the FTIR results (Figure 4b). Most of the CH_2_ rocking associated with the β-phase was located around 795 cm^−1^. Therefore, these results confirm the higher β-phase content in the present electrospun PVDF fibers than in the commercial powder form.

Furthermore, the relative−intensity ratios of two fixed wavelengths, at 1072 and 1276 cm^−1^ for the β-phase, were A = 1.02, B = 1.00 and C = 0.98. The highest value in sample A suggests the highest β-phase content amongst the three samples of different concentrations investigated in this study.

**Figure 7:**
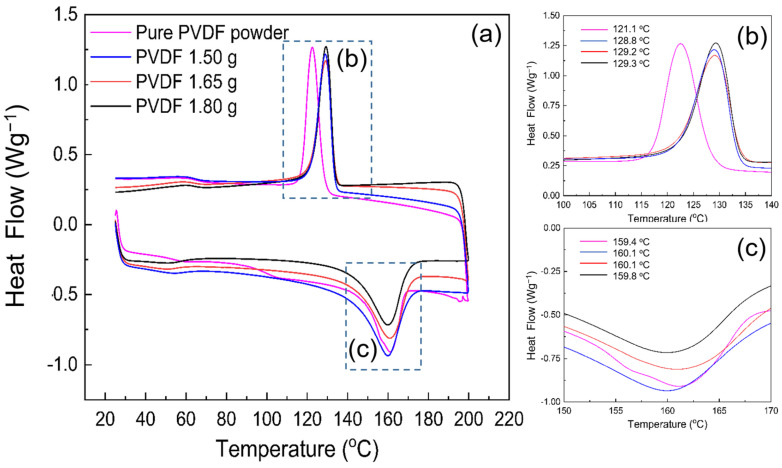
(**a**) DSC trace of the pure PVDF powder and electrospun PVDF nanofibers including samples A, B and C; (**b**) cooling thermograms and (**c**) heating thermograms of fibrous and powdery samples.

The differential scanning calorimetry (DSC) thermograms of electrospun PVDF nanofibers for samples A, B, C and the pure PVDF powder are shown in Figure 7. The peaks around 155 to 165 °C were similar for all samples; however, minor differences in the melting temperatures were still visible, which are summarized in Table 3 in the thermal-analysis results. In the first heating scan, the broad melting endotherms observed for the electrospun nanofibers are in agreement with the literature, and all electrospun samples showed a melting endotherm, confirming the predominant existence of the β-phase [22,42,43,44,45,46]. Conversely, for the powder sample, there was a small amount of α-phase present, as revealed by both XRD and FTIR analyses. Although the effect of the concentration on the crystallinity of PVDF was multifaceted, the results indicate that the degree of crystallinity of electrospun fibers was the highest for sample A. Herein, a shift to a lower temperature upon β-phase formation was observable for all samples except for sample C, and the melting temperature (T_m_) moved to a slightly higher temperature for sample C. The cooling curves were also affected by the variation of PVDF concentration, and the slight differences between their crystallization temperatures (T_c_) are summarized in Table 3. For the pure PVDF powder, the melting temperature peak was similar to that of sample C, whereas its 121.1 °C T_c_ was slightly lower than that of the electrospun nanofibers (Figure 7). The area of the melting peak increased when the concentration surged from sample A to C. The 2% increase in the crystallinity of the electrospun fibers for sample A, compared with the powder sample from 44.3 to 45.1, was significantly higher than the rest of the samples, which is due to the thinner fibers that formed. Additionally, a drop of almost 12% in crystallinity for samples B and C from 39.7 to 39.1 can be attributed to the shorter solidification period in the electrospinning process. When the loading of PVDF ranges from 15 to 18 wt%, the crystallinity of PVDF chains becomes low and the crystal ability of PVDF will be reduced. Similarly, this can be ascribed to the chain-motion restriction of PVDF chains of higher concentration than the moderate concentration of sample A [44].

### 3.3. Piezoelectric Response of Electrospun PVDF Fibers

**Figure 8:**
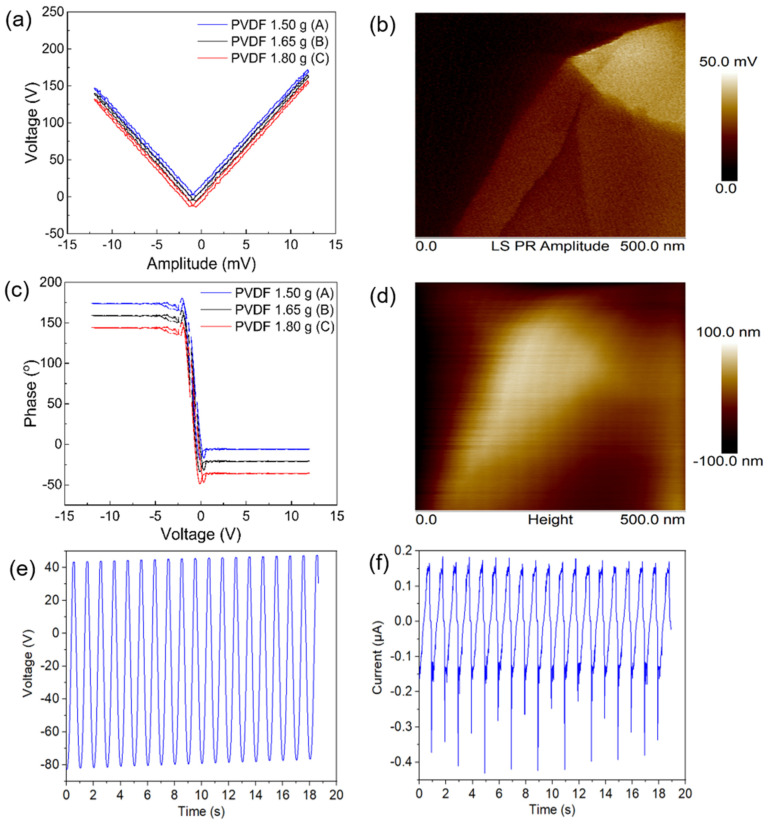
(**a**–**f**) Piezoresponse hysteresis loops for samples A, B and C and piezoresponse force microscope (PFM) phases and corresponding amplitude hysteresis in response to the applied voltage bias for samples A, B and C, and 7 (**e**,**f**) electrical response of electrospun PVDF nanofibers mat with 15% wt. PVDF under applied force of 50 N.

The local amplitude and the piezoresponse phase of the electrospun samples are shown in Figure 8a–d. A strong piezoelectric contrast was seen in the amplitude image because of the deflection generated by the applied AC field. The negative and positive values of the phase image for the nanofiber are due to the antiparallel ferroelectric nano domains with 180° domain walls for all the electrospun nanofiber samples. Under the effect of electric fields, the local hysteresis loops are shown in Figure 8a,b. The forward and backward strong voltages were −12.5 and 12.5 V. The captured data from the PFM images indicate that the magnitude of the amplitude is ~170 mV for sample A at E = 11.5 V, which was the highest amplitude of the piezoelectric signal among all the samples, and its amplitude and maps of surface-height images are shown in Figure 8c. As is clear from this figure, the phase versus applied voltage for samples A, B and C exhibited an almost 180° switching hysteresis loop as well as a complete switch in the polarization of each sample, as a result of the stress that was generated by the PFM tip.

Furthermore, the highest voltage and current outputs obtained from a square copper sheet of 30 × 30 mm^2^ coated by 0.015 mm thickness of electrospun PVDF nanofiber facing to the PET sheet attached to another square copper sheet of 30 × 30 mm^2^, were 124 V and 174 nA, which are plotted as Figure 8e,f. The effect of piezoelectricity can be seen as the transition from the α-phase of the powder to the β-phase of the electrospun fibers. The principle of tip-link activation is to deform them as a static nanofiber membrane through acoustic force, which is similar to the current investigation applied by PFM.

**Figure 9:**
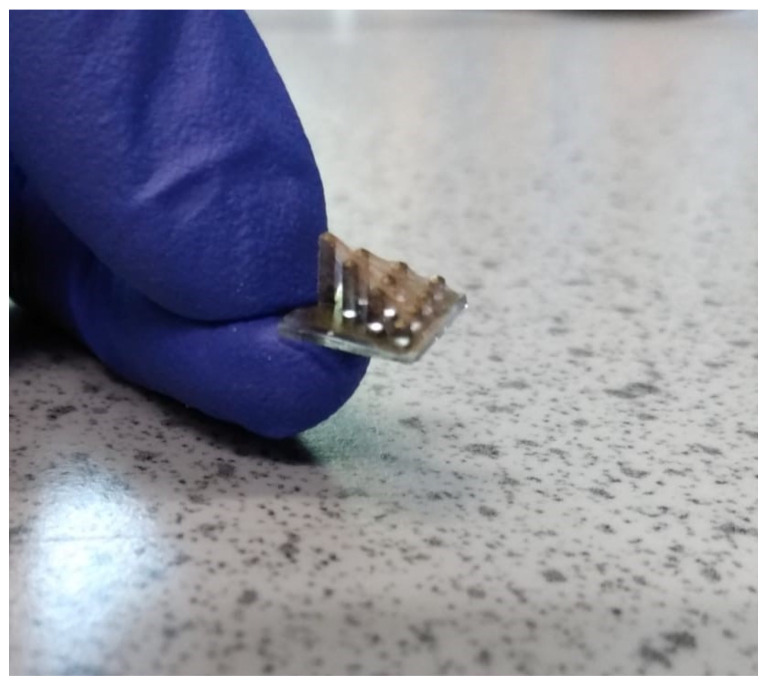
A prototype of the device including electrospun PVDF nanofibers mimicking the tip-link behavior on top and PDMS pillars substrate.

The above results highlight that the use of appropriate electrospun PVDF nanofibers of pure, biocompatible and flexible material can generate an open-circuit voltage (V_oc_) of 170 mV for a single fiber. This value can be sufficient to mimic artificial tip links, whereas the natural hair cell’s output can only produce 125 mV (V_oc_) [45]. Also, the mat of electrospun PVDF can conduct more piezoelectric charges to the surface of an attached device, as shown in a prototype using the electrospun mat, due to the tip links on the surface of vertical pillars mimicking the artificial hair cells, as shown in Figure 9. The PVDF connecting sequential tips of the pillars in the artificial hair cells mimics the biological tip links containing the main sensors in the superficial neuroma sensors in blind cavefish [46].

To develop the new generation of self-powered hair-cell sensors, PVDF cantilever beams can be used to construct the cochlear implants, enabling a self-powered device to sense the mechanical vibrations. These natural tip links as mechanoelectrical transducer (MET) channels are the main parts of auditory hair cells in the cochlea that convert the mechanical stimuli from sound into electrical signals. To restore mechanoelectrical transduction (MET) function of the tip links in hair cells, electrospun PVDF fibers fabricated in this investigation have proved promising.

## 4. Conclusions

Different concentrations of PVDF powder were used to create electrospun fibers of different diameters. The electrospinning parameters have an effect on the final structures and performance of the electrospun fibers. Among all the samples, sample A, with the minimum mean size of 213 nm and the highest β-phase content, generated the best piezoelectric properties, with V_oc_ (open-circuit voltage) of 170 mV, which is sufficient to function as a tip link. We further confirmed that the highest piezoelectric voltage and current outputs from a thickness of 0.015 mm of electrospun PVDF fibers were 124 V and 174 nA, respectively. This fundamental study, plus the constructed prototype, demonstrated that electrospun PDVF nanofibers can be engineered to function as artificial tip links in the cochlea sensor to link the artificial hair cells in the construction of the next generation of self-powered artificial hearing aids.

## Figures and Tables

**Table 1:** The elemental compositions (in atomic percentage) of the pristine PVDF powder and the as-prepared fiber samples.

Samples	C 1s (At%)	O 1s (At%)	F 1s (At%)	F/C	O/C
Pure PVDF powder	54.64	1.77	43.59	0.80	0.032
Sample A	59.58	1.78	38.65	0.65	0.030
Sample B	55.33	0.85	43.82	0.79	0.015
Sample C	57.30	1.04	41.66	0.73	0.018

**Table 2:** Atomic percentage of C 1s surface chemical composition of the pristine PVDF powder and the as-prepared fiber samples.

Samples	CF_2_ (At%)	CH_2_ (At%)	-COO (At%)	CH (At%)	CF_3_ (At%)
Pure PVDF powder	20.22	26.35	2.89	3.04	0.48
Sample A	19.04	23.51	3.08	11.99	0.36
Sample B	20.62	22.19	2.68	6.12	0.30
Sample C	20.74	23.59	2.82	6.67	0.74

**Table 3:** DSC analysis of the pure PVDF powder and electrospun fibers, samples A, B and C. T_m_: Melting temperature; ∆H_m_: enthalpy of fusion and X_DSC_: degree of crystallinity.

Samples	T_c_ [°C]	T_m_ [°C]	∆H_m_ [J.g^−1^]	X_DSC_
Pure PVDF powder	121.1	160.1	45.2	44.2
Sample A	128.8	159.4	46.7	45.1
Sample B	129.2	159.8	43.3	39.7
Sample C	129.3	160.1	41.1	39.1

## Data Availability

Not applicable.

## Supplementary Materials

The following supporting information can be downloaded at: https://www.mdpi.com/article/10.3390/nano12091466/s1, Figure S1: SEM images of the electrospun (a) sample A, (b) sample B and (c) sample C prepared at 15 cm between the needle and collector at the same feeding rate of 3 mL h^−1^, voltage of 15 kV and collector speed of 1200 rpm, and (d) the relationship between the mean fiber diameter (nm) vs. concentration of PVDF (wt.%); Figure S2: SEM images of samples as (a) sample D, (b) sample E and (c) sample F, under three different voltage values for 15 (wt.%) of PVDF and 15cm distance between the needle and collector in the same feeding rate of 3 mL h^−1^ and the collector speed of 1200 rpm, and (d) the relation between the mean fiber diameter (nm) vs. voltage values (kV.); Figure S3: SEM images of samples as (a) sample G, (b) sample H and (c) sample I, for three different distance values for 15(wt.%) of PVDF under 20 kV voltage value in the same feeding rate of 3 mL h^−1^ and the collector speed of 1200 rpm, and (d) the relation between the mean fiber diameter (nm) vs. distance from needle to the rotary collector (cm); Figure S4: SEM images of samples as (a) sample J, (b) sample K and (c) sample L, for three different feeding rates for 15 (wt.%) of PVDF under 20 kV voltage value and the collector speed of 1200 rpm, and (d) the relation between the mean fiber diameter (nm) vs. feeding rate (mL h^−1^); Figure S5: SEM images of samples as (a) sample M, (b) sample N and (c) sample O, for three different collector speed rates for 15 (wt.%) of PVDF under 20 kV voltage value in the same feeding rate of 3 mL h^−1^, and (d) the relation between the mean fiber diameter (nm) vs. collector speed (rpm); Figure S6: The schematic of materials order for the triboelectric property of PVDF electrospun samples; Figure S7: Fraction of β-phase in percentage vs. the concentration of PVDF solution. Table S1: The effect of PVDF concentration on the mean diameter of the nanofibers; Table S2: The effect of changing voltage value on the mean nanofiber diameter; Table S3: The effect of changing distance between the needle and the collector value on the mean nanofiber diameter size; Table S4: The effect of changing feeding rate value on the mean nanofiber diameter size; Table S5: The effect of changing collector speed value on the mean nanofiber diameter size; Table S6: The effect of changing concentration of PVDF in the fraction content of β-phase. References [47,48,49,50,51,52] are cited in the Supplementary Materials.
