# Supplementary material for: Piezoelectric Property of Electrospun PVDF Nanofibers as Linking Tips of Artificial-Hair-Cell Structures in Cochlea
Source: Nanomaterials (Basel). 2022 Apr 26;12(9):1466. doi: 10.3390/nano12091466 (PMC9104576; doi:10.3390/nano12091466)
Supplement: Supplementary file 1 [file nanomaterials-12-01466-s001.zip › nanomaterials-1678417-supplementary.pdf]

Supplementary materials

Piezoelectric Property of Electrospun PVDF Nanofibers as Linking Tips of Artificial-Hair-Cell Structures in Cochlea

Rana Sabouni Tabari [1], Yu Chen [1,*], Kunyapat Thummavichai [1], Yan Zhang [2], Zakaria Saadi [1], Ana I. S. Neves [1], Yongde Xia [1] and Yanqiu Zhu [1,*]

[1] Department of Engineering, University of Exeter, Exeter, EX4 4SB, UK; rs683@exeter.ac.uk (R.S.T), kt302@exeter.ac.uk (K.T), z.saadi@exeter.ac.uk (Z.S), a.neves@exeter.ac.uk (A.I.S.N), y.xia@exeter.ac.uk (Y.X)

[2] State Key Laboratory of Powder Metallurgy, Central South University, Changsha 410083, China; 219120@csu.edu.cn;

* Correspondence: yc465@exeter.ac.uk (Y.C.); y.zhu@exeter.ac.uk (Y.Z.)

1. SEM characterizations for proposed parameters on achieved PVDF electrospun structures

1.1 The effect of concentration on morphology

Table S1 summarizes the effect of PVDF concentration on the mean nanofibers size, tested while other parameters kept constant, including voltage, spray distance, and feeding rate. According to the mean diameter, the thinnest fibers were achieved when using the lowest concentration of PVDF. Figure S1(a−c) shows the SEM images of the three different electrospun PVDF nanofibers, produced using parameters listed in Table S1. Sample A produced the best result, with a the mean nanofiber diameter of 270 nm, and quite uniform. In contrast, for samples B and C, the mean diameters increased significantly to 583 and 989 nm, separately, also became un-uniform. As the diameters are directly affected by the solution concentration, a good distribution on the surface of fibers is achieved only at proper PVDF concentration. For concentration higher than 15 wt.%, the formation of beaded and large nanofibers is evident. However, when the concentration was lower than 12 wt.%, unspinnable products resulted, in the form of droplets instead of nanofibers. At the proper concentration, the diameter of the fibers will be in the sub-micron scale, and the distribution will be more homogenous and even.

Table S1 The effect of PVDF concentration on the mean diameter of the nanofibers.

Samples	Solvent	Concentration (wt.%)	Voltage (kV)	Distance (cm)	Feeding rate (mL h^{-1})	Collector speed (rpm)	Mean nanofiber diameter (nm)
A	DMF/Acetone	15	20	15	3	1200	270
B	DMF/Acetone	16.5	20	15	3	1200	583
C	DMF/Acetone	18	20	15	3	1200	989

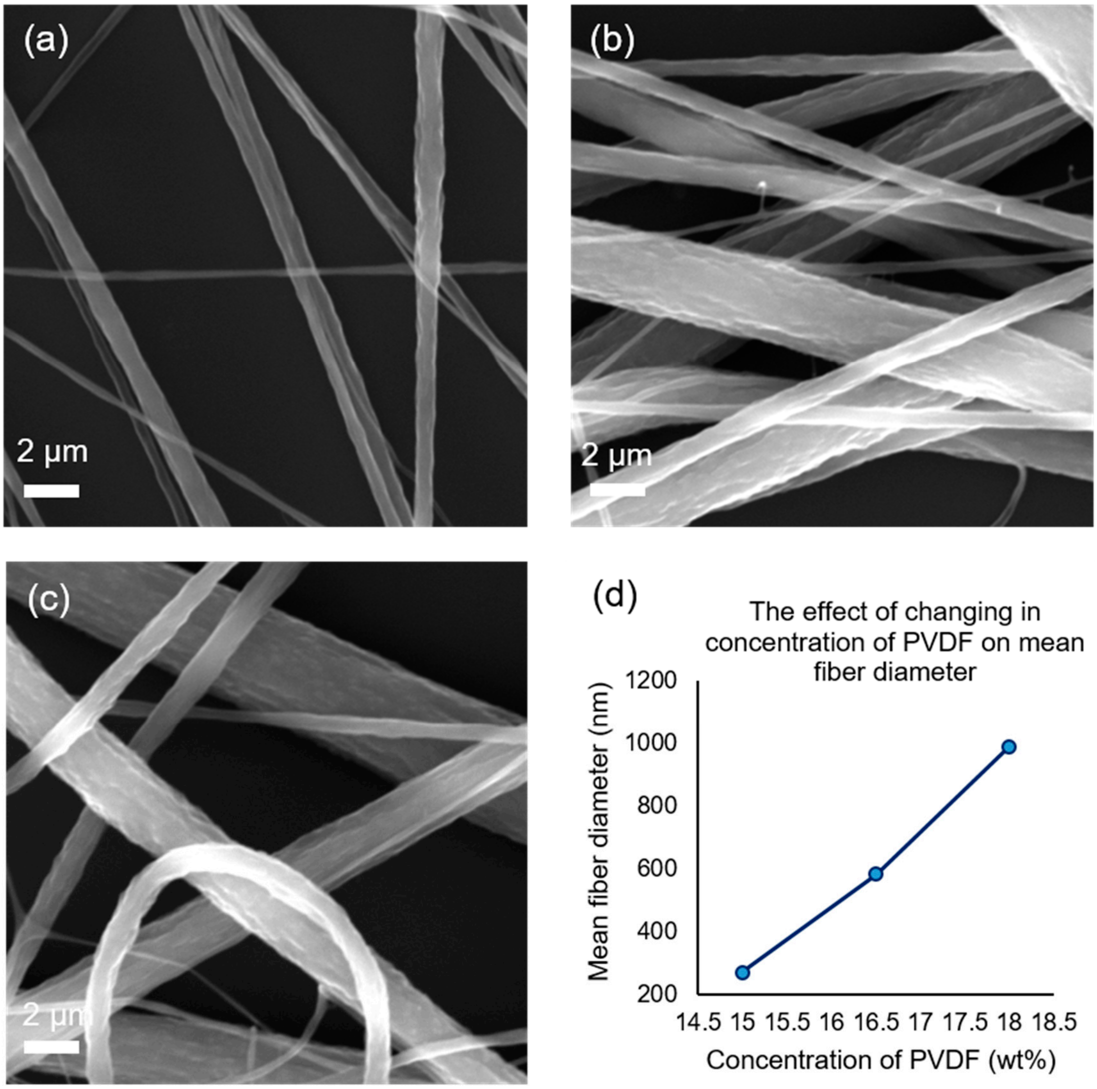

Figure S1 SEM images of the electrospun (**a**) sample A, (**b**) sample B and (**c**) sample C prepared at 15 cm between the needle and collector at the same feeding rate of 3 mL h^{-1}, voltage of 15 kV and collector speed of 1200 rpm, and (**d**) the relationship between the mean fiber diameter (nm) vs concentration of PVDF (wt.%).

1.2 The effect of changing in voltages on morphology

Table S2 summarizes the parameters used to investigate the voltage effect on the morphology of the electro spun fibers, under a constant distance of 15 cm between the needle and the collector, a feeding rate of 3 mL h^{-1} and collector rotating speed of 1200 rpm. At 20 kV, a mean fiber diameter of 268 nm was obtained (Figure S2(b)); whilst at 15 kV, the mean diameter was reduced to 245 nm but accompanied with beads and bending fibers, as shown in (Figure S2(a)). At 25 kV, the fibers displayed a sudden change in areas, also accompanied with beads and bending, with a mean fiber diameter of 213 nm. However, non-alignment and poorly distributed distribution became sever at 25 kV, as shown in Figure S2(c). While some argue that the increase in voltage value will lead to an increase in diameter size regarding the type of solutions, some might believe that the greater voltage values could lessen the final size of diameter due to the increase in electrostatic repulsive force [1].

The ejection of solution initiation happens when the voltage value is as much as to overcome the surface tension in the polymer solution. Nevertheless, there is no specific difference among the diameter values of these fibers in three different voltage values, but fewer fibers are in lower voltage values while they get extremely intensive in higher voltage values, which could be due to the higher amount of polymeric solution charging into the collector. Herein, the increase in electrostatic forces of the PVDF solution, leads to the fiber diameter decrease. The change of α-phase to the dominant β-phase occurs due to the high voltage applicator on the stretched polymer jet [2]. For the current experimental condition, the optimum value of applied voltage is 20 kV which might differ from other studies [3,4]. As the voltages exceeds the mentioned value, less intensity of β-phase content and crystallinity of PVDF will occur.

Table S2 The effect of changing voltage value on the mean nanofiber diameter.

Samples	Solvent	Concentration (wt.%)	Voltage (kV)	Distance (cm)	Feeding rate (mL h^{-1})	Collector speed (rpm)	Mean nanofiber diameter (nm)
D	DMF/Acetone	15	15	15	3	1200	245
E	DMF/Acetone	15	20	15	3	1200	268
F	DMF/Acetone	15	25	15	3	1200	213

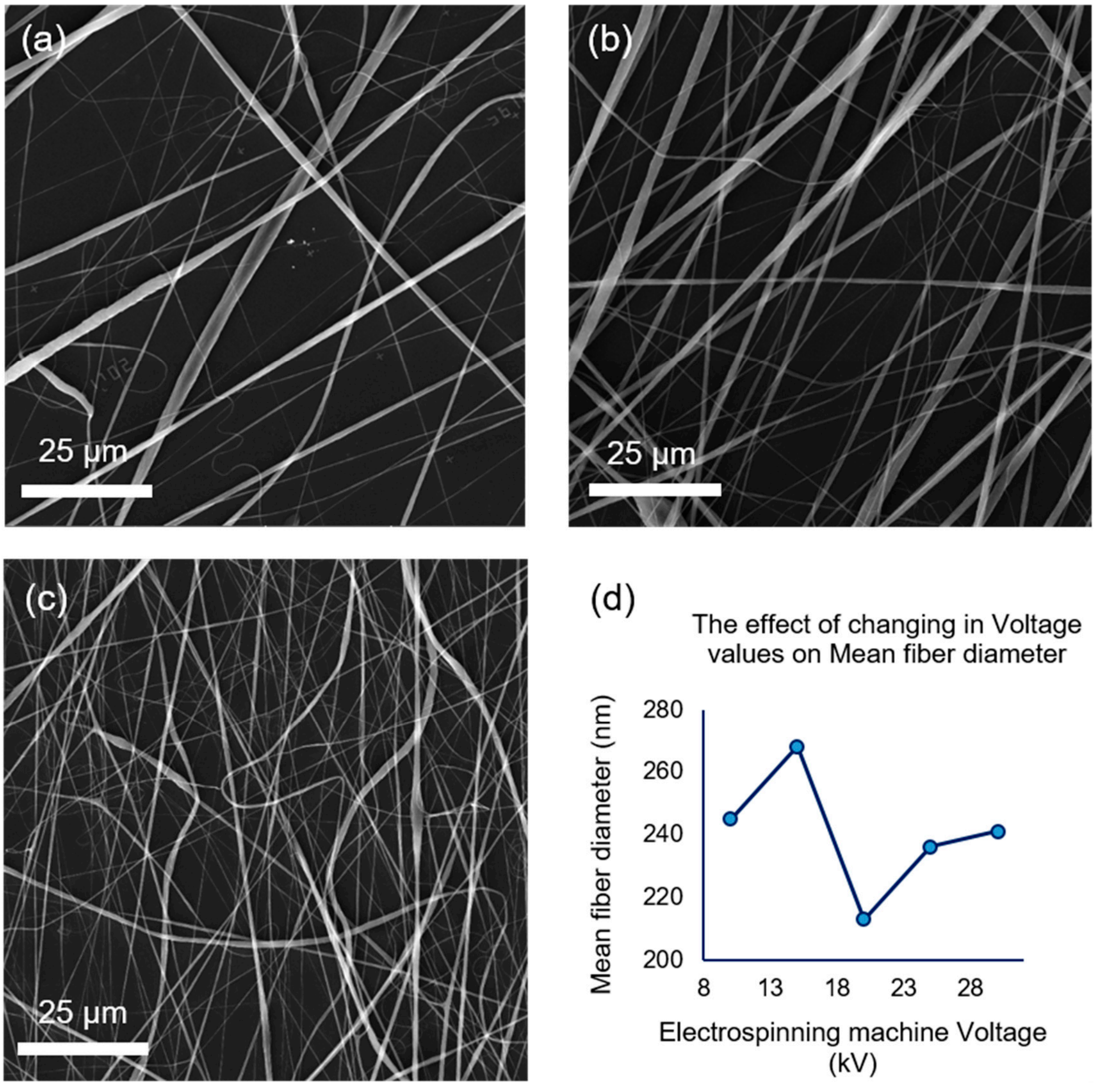

Figure S2 SEM images of samples as (**a**) sample D, (**b**) sample E and (**c**) sample F, under three different voltage values for 15 (wt.%) of PVDF and 15cm distance between the needle and collector in the same feeding rate of 3 mL h^{-1} and the collector speed of 1200 rpm, and (**d**) the relation between the mean fiber diameter (nm) vs voltage values (kV.).

1.3 The effect of changing the distance between the needle and the collector on morphology

Some of the previous studies declared that there is no significant change in the relative decrease in the diameter values of fibers [5–7], however herein a linear relationship is seen.

Table S3 indicates one of the most influential factors on the electrospinning process as the evaporation of solvent from the nanofibers. The time which helps for less defect fibers fabrication is relevant to the distance of the needle and the collector in this methodology. For this reason, three different electrospinning distance conditions on the morphology of the final results are investigated which are summarized in Table S3 and illustrated as SEM images (a) sample : 10 cm, (b) sample : 15 cm and for (c) sample : 20 cm). For longer distances than 10 cm, the mean diameter has decreased.

The SEM results show a less bead and branched electrospun nanofibers and the analysis of diameter measurement is more reliable due to the semi equal distribution of the same nanofibers' diameters. Any decrease in the distance between the needle tip and the collector makes beads and branches possible among nanofibers and the average diameter reaching will not be easy (Figure S3(a—c)). After several analyses of the electrospinning process, the average diameter of 250—270 nm is almost satisfactory for being appropriate in all of the parameters presented in the study. The below results including the SEM images and Table S3 confirm that if the distance is getting fallen, a major increase in the size of the fibers' diameter will happen. To fabricate bead-free and non-branched nanofibers the more modified and appropriate polymeric solution is essential. While the fabrication of fibers for 1.50 g of PVDF in 10 ml ACE/DMF solution (15 wt.%) is easy, the fabrication of PVDF electrospun fibers for more than 1.80 g, is very difficult. Hence, the electro spin ability of each solution is different from the other ones.
As the distance between the needle-tip to the collector is increasing, the web collection of fibers is changing in the average diameter of fibers. For distances less than 15 cm, the shape of fibers is less circular, however, the more the distance is changing from 15 cm, the shape of fibers is core-shell. The mean diameter of electrospun nanofibers is around 310 nm as the distance is 10 cm (Figure S3(a)). Interestingly, the mean diameter of electrospun nanofibers has decreased to 209 nm when the distance gets risen, however less aligned nanofibers and more bends could be seen. As a consequence, for the best distance between the needle and the collector (15 cm).

Table S3 The effect of changing distance between the needle and the collector value on the mean nanofiber diameter size.

**Samples**	**Solvent**	**Concentration (wt.%)**	**Voltage (kV)**	**Distance (cm)**	**Feeding rate (mL h^{-1})**	**Collector speed (rpm)**	**Mean nanofiber diameter (nm)**
G	DMF/Acetone	15	20	10	3	1200	310.
H	DMF/Acetone	15	20	15	3	1200	258.
I	DMF/Acetone	15	20	20	3	1200	209.

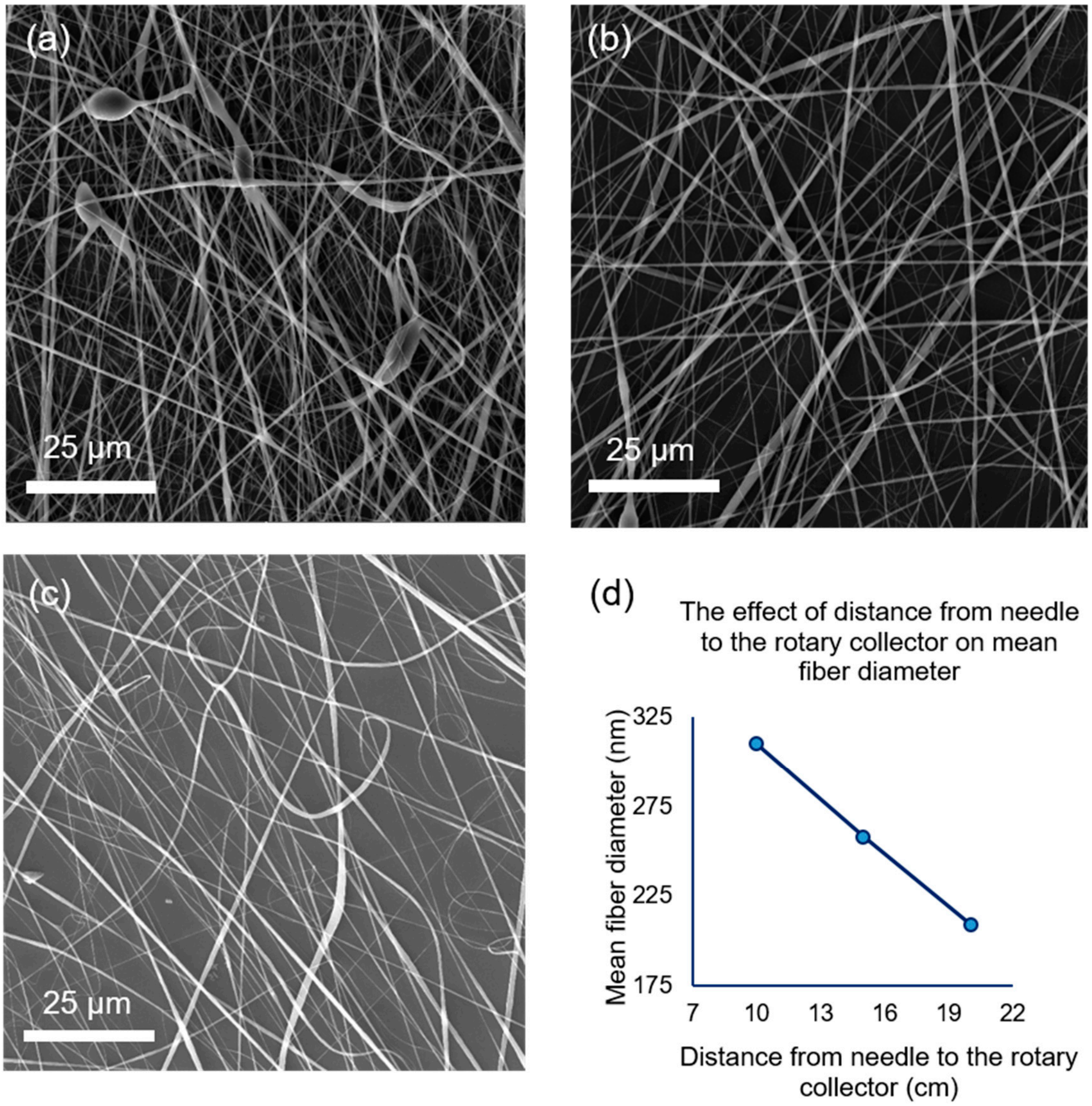

Figure S3 SEM images of samples as (**a**) sample G, (**b**) sample H and (**c**) sample I, for three different distance values for 15(wt.%) of PVDF under 20 kV voltage value in the same feeding rate of 3 mL h^{-1} and the collector speed of 1200 rpm, and (**d**) the relation between the mean fiber diameter (nm) vs distance from needle to the rotary collector (cm).

1.4 The effect of changing the solution feeding rates on morphology

The condition parameters of three different feeding rate samples (samples J, K and L) were shown in Table S4 and the result of their morphological evolution is presented in Figure S4. As it is shown, for the feeding rates equal to 5 mL h^{-1}, the mean diameter size is increased by 59.80 %. Compared with 1 mL h^{-1}, feeding rate.
As SEM and TEM images illustrate, for the lowest flow rate value which is 1 mL h^{-1}, the whole morphology of electrospun nanofibers is different. An increase in the electrical current happened when the feeding rate values increased. While the fiber diameter is less for the feeding rate of 1 mL h^{-1}, beads are possible to be seen and the mean nanofiber diameter is the minimum size and is 202 nm, however, when the feeding rate is 3 mL h^{-1},no beads could be seen and the mean nanofiber diameter is 258 nm and TEM result approves the lowest diameter size available in this feeding rate as well. For the lower feeding rates than 3 mL h^{-1}, the polymeric solution detaches out of the needle tip to the collector by the electric force, while for the higher feeding rate than 3 mL h^{-1}, the faster injection leads to more beads formation and less uniform morphology. Also, the thicker fibers are

when the feeding rate is 5 mL $h^{-1}$with an increase of 48.70% in the mean nanofibers diameter size. Although in some areas for the highest feeding rate, electrospun nanofibers are partly aligned, however when the feeding rate is 3 mL h^{-1}, the electrospun nanofibers seem more even. Therefore, at the mentioned given conditions, for 3 mL h^{-1} feeding rate, the morphology is more constant and unvarying.

The representative TEM image of the PVDF electrospun nanofibers, a uniform and pure structure of fiber is observable and the surface morphology of nanofibers is nearly even in most parts of aligned nanofibers. The structure is core-shell and the dark region allocated to core structure and the bright region is shell.

Table S4 The effect of changing feeding rate value on the mean nanofiber diameter size.

Samples	Solvent	Concentration (wt.%)	Voltage (kV)	Distance (cm)	Feeding rate (mL h^{-1})	Collector speed (rpm)	Mean nanofiber diameter (nm)
J	DMF/Acetone	15	20	15	1	1200	202
K	DMF/Acetone	15	20	15	3	1200	258
L	DMF/Acetone	15	20	15	5	1200	480

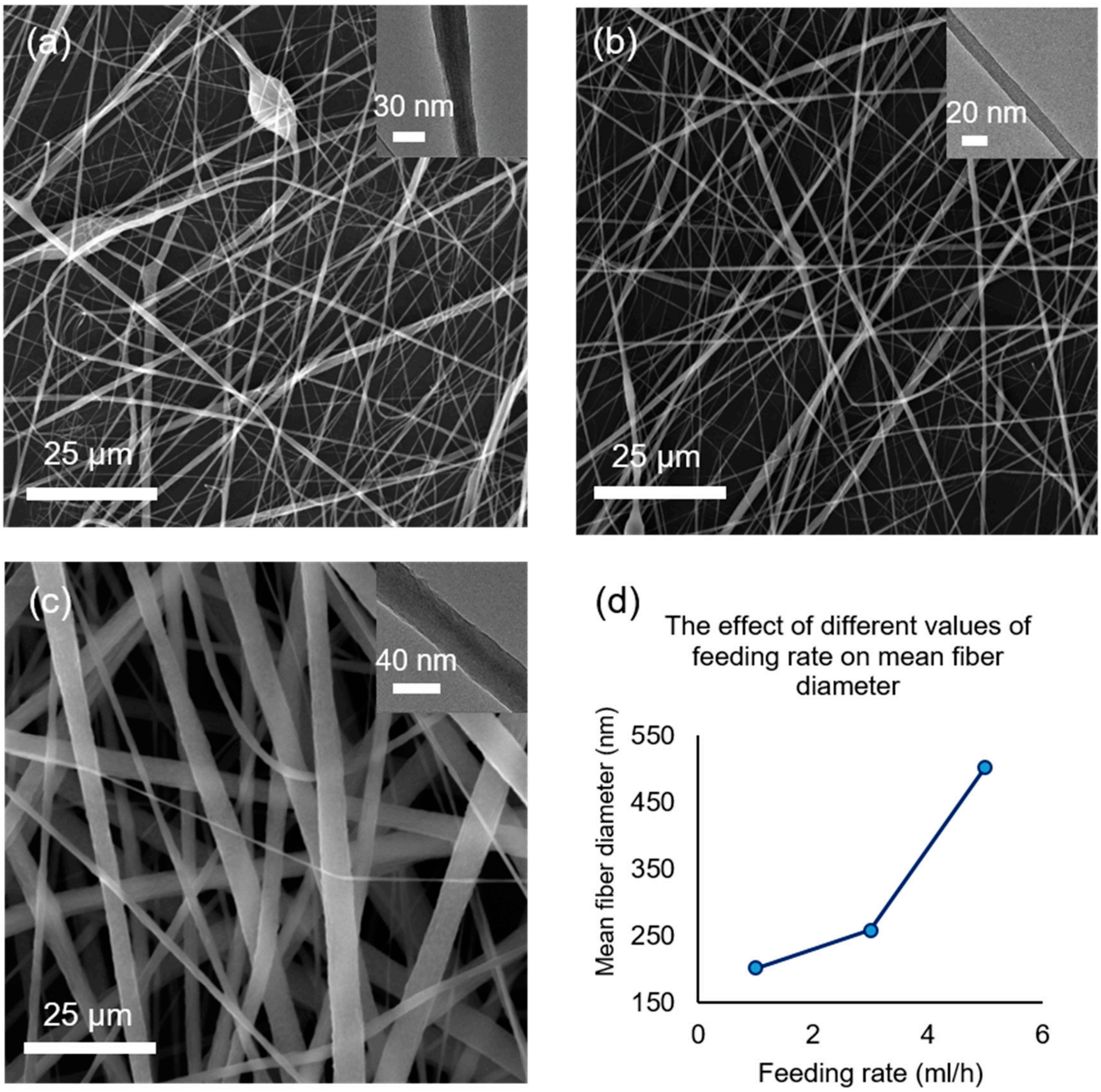

Figure S4 SEM images of samples as (**a**) sample J, (**b**) sample K and (**c**) sample L, for three different feeding rates for 15 (wt.%) of PVDF under 20 kV voltage value and the collector speed of 1200 rpm, and (**d**) the relation between the mean fiber diameter (nm) vs feeding rate (mL h^{-1}).

1.5 The effect of changing the speed rate of collector on morphology

The highest speed rate of the collector in our set-up is almost 1300 rpm and for the current experiment, the most appropriate and safest speed to achieve better PVDF electrospun nanofibers was 1200 rpm.

Table S5 summarizes the effect of an increase in the value of speed rates on the size and distribution of PVDF electrospun PVDF nanofibers. As it is well defined, for the highest value of the collector speed, the diameter of nanofibers has been decreased from 368 nm to 271 nm.

As the SEM images show, the lower the speed, the thicker the fibers could be presented. When the speed is the lowest and equal to 400 rpm, the mean nanofiber diameter value is 368 nm and they are not aligned, somehow tied together and crossed. Even for an increment of 400 to the collector speed, the mean nanofiber diameter value has not changed significantly as summarized in Table S5 and the value of the mean nanofiber diameter value is 308 nm. A decrease of almost 30% in the diameter size of the PVDF nanofibers is evident when the speed of the collector is increasing from 800 rpm to 1200 rpm. The most appropriate value of speed rate of collector is 1200 rpm for which there are no more beads and non-aligned electrospun nanofibers existing, but

also the mean diameter size is close to the other proper values in previous SEM characterizations for different parameters investigations.

Table S5 The effect of changing collector speed value on the mean nanofiber diameter size.

Samples	Solvent	Concentration (wt.%)	Voltage (kV)	Distance (cm)	Feeding rate (mL h−1)	Collector speed (rpm)	Mean nanofiber diameter (nm)
M	DMF/Acetone	15	20	15	3	400	368
N	DMF/Acetone	15	20	15	3	800	308
O	DMF/Acetone	15	20	15	3	1200	271

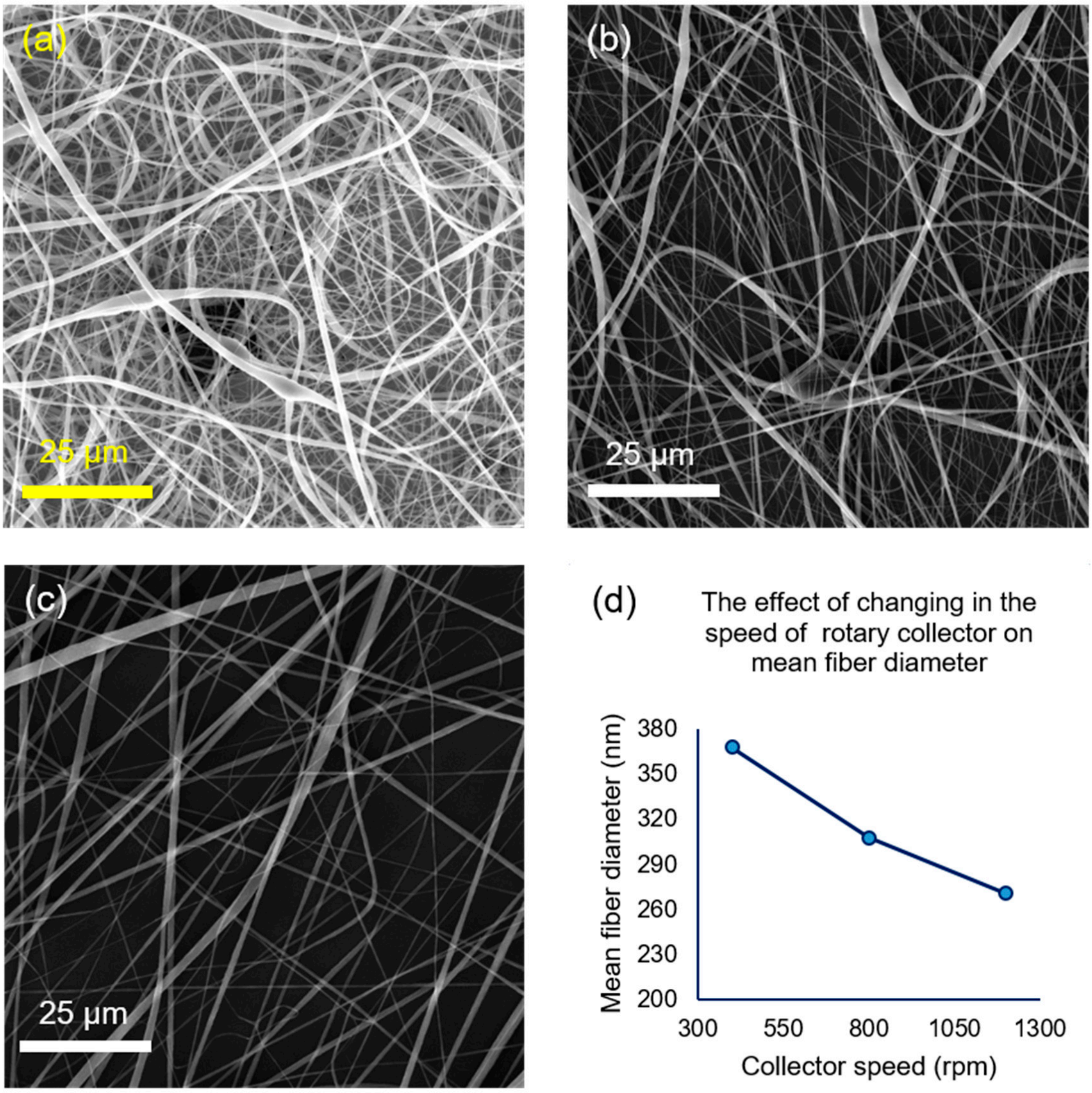

Figure S5 SEM images of samples as (**a**) sample M, (**b**) sample N and (**c**) sample O, for three different collector speed rates for 15 (wt.%) of PVDF under 20 kV voltage value in the same feeding rate of 3 mL h−1, and (**d**) the relation between the mean fiber diameter (nm) vs collector speed (rpm).

Very briefly, SEM images in Figure S1(a-c) and Table S1 show the increase of more than 115 % in mean diameter after an escalating in the concentration of PVDF in DMF from 15 to 16.5 wt.% for samples A and B. Following this surge, sample C shows the thickest PVDF electrospun fiber and 266 % rise in the diameter compared with sample A. As shown in Figure S2(a-c) and Table S2, 20 kV has been considered as the suitable voltage for the electrospinning of PVDF nanofibers, 15 cm is the ideal distance between the needle and the collector (Figure S3(a−c) and Table S3), 3 mL h^{-1} the optimal feeding rate (Figure S4(a−c) and Table S4), and at a collecting drum rate of 1200 rpm (Figure S5(a−c) and Table S5). Further increase in the feeding rate would lead to an observable change in the fiber diameters.

2.The preparation of the electrospun device for the acoustic linear characterization

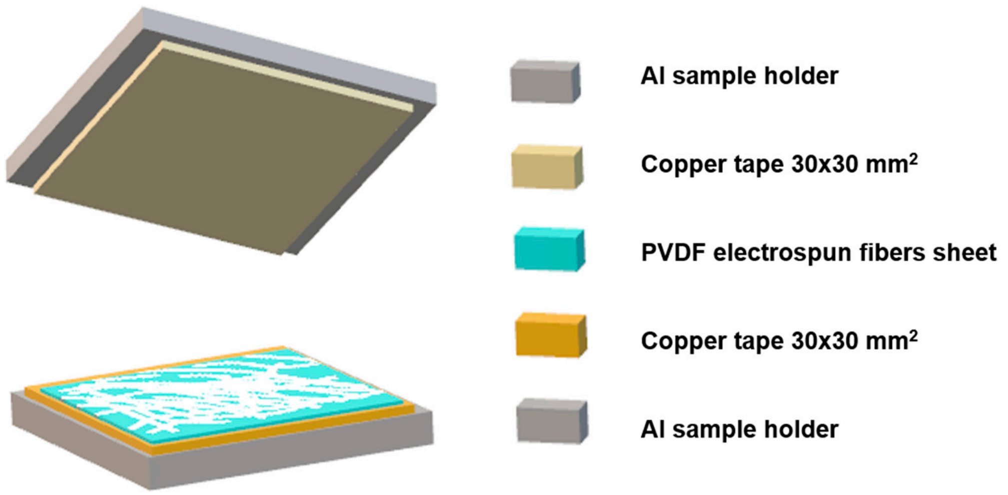

Figure S6 The schematic of materials order for the triboelectric property of PVDF electrospun samples.

3. The quantification of β-phase in the electrospun fibers by FTIR results

The simplified form of the Lambert Beer law is used to quantify the phase contents in PVDF electrospun fibers. Assuming absorption coefficients as $K\alpha = 6.1 \times 10^4\ cm^2mol^{-1}$ and $K\beta = 7.7\times 10^4\ cm^2mol^{-1}$ for respective wavenumbers of 766 and 840 cm^{-1} in all electrospun samples A, B and C, the equation will be expressed as below [8].

$$F(\beta) = \frac{A_\beta}{\left(\frac{K_\beta}{K_\alpha}\right) A_\alpha + A_\beta} \quad (1)$$

With the objective to achieve the highest fraction of β-phase, the best range of electrospinnable production relevant to optimized range of concentration starting from 15 to 18 wt.%, is summarized in Table S6 and the F(β) vs the change in concentration is illustrated as Figure S7. The increase in concentration helps the enhancement in β fraction value, however, it starts decreasing from the concentration more than 15 wt.%. Effect of lower concentrations will be presented elsewhere.

Table S6 The effect of changing concentration of PVDF in the fraction content of β-phase.

Samples	Solvent	Concentration (wt.%)	A_α	A_β	F(β)
A	DMF/Acetone	15	0.173	0.844	0.89
B	DMF/Acetone	16.5	0.124	0.872	0.79
C	DMF/Acetone	18	0.234	0.701	0.70

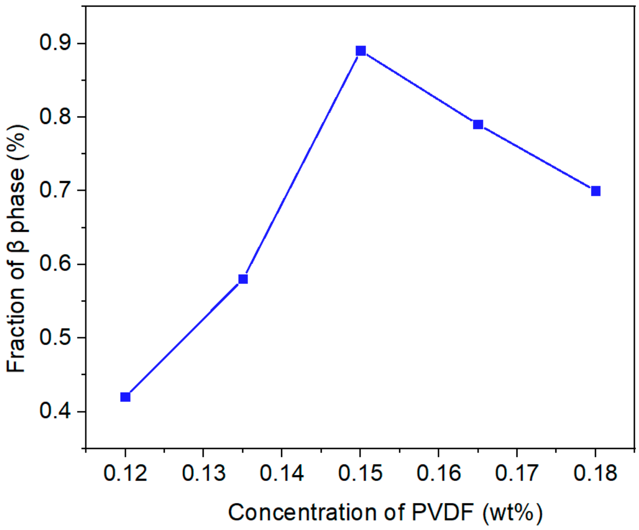

Figure S7 Fraction of β-phase in percentage vs. the concentration of PVDF solution.

References

1. Sultana, T.; Georgiev, G.L.; Auner, G.; Newaz, G.; Herfurth, H.J.; Patwa, R.; XPS analysis of laser transmission micro-joint between poly (vinylidene fluoride) and titanium. *Appl. Surf. Sci.*, **2008**, *255*, 2569–2573. https://doi.org/10.1016/j.apsusc.2008.07.149.
2. Sajkiewicz, P.; Wasiak, A.; Goclowski, Z. Phase transitions during stretching of poly(vinylidene fluoride). *Eur. Polym. J.* **1999**, *35*, 423–429. https://doi.org/10.1016/S0014-3057(98)00136-0.
3. Mazoochi, T.; Hamadanian, M.; Ahmadi, M.; Jabbari, V. Investigation on the morphological characteristics of nanofiberous membrane as electrospun in the different processing parameters. *Int. J. Ind. Chem.*, **2012,** *3*, 1–8. https://doi.org/10.1186/2228-5547-3-2.
4. Can-Herrera, L.A.; Oliva, A.I.; Dzul-Cervantes, M.A.A.; Pacheco-Salazar, O.F.; Cervantes-Uc, J.M. Morphological and mechanical properties of electrospun polycaprolactone scaffolds: Effect of applied voltage. *Polymers*, **2021**, *13*, 1–16. https://doi.org/10.3390/polym13040662..
5. Nasir, M.; Matsumoto, H.; Danno, T.; Minagawa, M.; Irisawa, T.; Shioya, M.; Tanioka, A.; Control of diameter, morphology, and structure of PVDF nanofiber fabricated by electrospray deposition. *J. Polym. Sci. Part B Polym. Phys.*, **2006**, *44*, 779–786. https://doi.org/10.1002/polb.20737.
6. Motamedi, A.S.; Mirzadeh, H.; Hajiesmaeilbaigi, F.; Bagheri-Khoulenjani, S.; Shokrgozar, M. Effect of electrospinning parameters on morphological properties of PVDF nanofibrous scaffolds. *Prog. Biomater.*, **2017**, *6*, 113–123. https://doi.org/10.1007/s40204-017-0071-0.
7. Bae, J.; Baek, I.; Choi, H. Efficacy of piezoelectric electrospun nanofiber membrane for water treatment. *Chem. Eng. J.*, **2017**, *307*, 670–678. https://doi.org/10.1016/j.cej.2016.08.125.
8. Gregorio, R.; Ueno, E.M. Effect of crystalline phase, orientation and temperature on the dielectric properties of poly (vinylidene fluoride) (PVDF). *J. Mater. Sci.* September **1999**, *34*, 4489–4500. https://doi.org/10.1023/A:1004689205706.
